# A Rare Case of Mandibular Chondrosarcoma Arising from Fibrous Dysplasia

**DOI:** 10.3390/dj13120560

**Published:** 2025-12-01

**Authors:** Ciprian Roi, Ruxandra Elena Luca, Alexandra Roi, Adrian Nicoară, Sorina Fanan, Flavia Zară, Raluca Maria Cloșca, Mircea Riviș

**Affiliations:** 1 Department of Anesthesiology and Oral Surgery, Research Center of Dento-Alveolar Surgery, Anesthesia and Sedation in Dental Medicine, “Victor Babes” University of Medicine and Pharmacy, Eftimie Murgu Sq. No. 2, 300041 Timisoara, Romania; ciprian.roi@umft.ro (C.R.); nicoara.adrian@umft.ro (A.N.); rivis.mircea@umft.ro (M.R.); 2 University Clinic of Oral Rehabilitation and Dental Emergencies, Faculty of Dentistry, Interdisciplinary Research Center for Dental Medical Research, Lasers and Innovative Technologies, “Victor Babes” University of Medicine and Pharmacy, Eftimie Murgu Square No. 2, 300041 Timisoara, Romania; 3 Department of Oral Pathology, Multidisciplinary Center for Research, Evaluation, Diagnosis and Therapies in Oral Medicine, “Victor Babes” University of Medicine and Pharmacy, Eftimie Murgu Sq. No. 2, 300041 Timisoara, Romania; 4 Clinical County Emergency Hospital Timisoara, Oral and Maxillofacial Surgery Deprtment, 5 Take Ionescu Street, 300062 Timisoara, Romania; butariuso@gmail.com; 5 Department of Pathology, Emergency City Hospital and Department of Microscopic Morphology, Faculty of Medicine, “Victor Babes” University of Medicine and Pharmacy, Eftimie Murgu Sq. No. 2, 300041 Timisoara, Romania; flavia.zara@umft.ro (F.Z.);

**Keywords:** fibrous dysplasia, malignant transformation, chondrosarcoma

## Abstract

Fibrous dysplasia is an uncommon, intramedullary fibro-osseous lesion representing approximately 5% to 7% of benign fibro-osseous lesions. The incidence of malignant transformation of fibrous dysplasia is considered very rare. The aim of this case report is to present a rare case of a chondro-sarcomatous transformation of a case previously diagnosed with mandibular fibrous dysplasia, a patient who was initially referred for a gingival tumoral extirpation in the third quadrant. A 57-year-old female patient presented for a consultation and, after a biopsy, was diagnosed with fibrous dysplasia of the mandibular bone. After two interventions of surgical removal of the lesion, the malignant transformation of chondrosarcoma occurred, which was identified by radiologic and histopathologic investigations, with the overall AJCC staging being pT1N0M0, G3. Radical resection of the mandibular bone with free margins of tumor was performed. Malignant transformation of mandibular fibrous dysplasia is a very rare and challenging complication of this disease. Early recognition and proper surgical treatment must be the key actions from doctors and clinicians.

## 1. Introduction

Fibrous dysplasia is an uncommon, intramedullary fibro-osseous lesion representing approximately 5–7% of benign fibro-osseous lesions [1,2]. It is a genetic non-inherited pathology caused by a missense mutation involving exon 8 of the Gsa gene on the long arm of the chromosome 20 at 20q13.2-3. This modification substitutes histidine, cysteine, or serine for arginine at position 201 within the alpha chain of the heterotrimeric G-subunit. Lichtenstein and Jaffe originally described it in 1942 as a disease characterized by the replacement of the medullary bone with immature and poorly calcified bone [3,4,5,6].

The clinical forms of fibrous dysplasia can be either the monostotic or polyostotic types. The monostotic type affects only one bone, unlike the polyostotic one, which has several systemic implications. In the case of McCune–Albright syndrome (MAS), it is characterized by polyostotic fibrous dysplasia, café-au-lait patches on the skin, and hormonal instability [7]. Monostotic fibrous dysplasia is the most prevalent type of fibrous dysplasia, and its localization is unilateral. Typically, it has a higher incidence in females and in 50% of cases of monostotic fibrous dysplasia target the bones of the head and neck area [8]. The most frequently affected areas belong to the craniofacial region, especially the mandible and maxilla, followed by the femur, tibia, and pelvis. As reported, the majority of lesions that affect the maxilla and mandible have a unilateral distribution, especially in the posterior area of these bones [9,10].

This pathology develops gradually, without early oral and maxillofacial symptoms, having a prolonged evolution, being easily overlooked by the patients. As the condition has a slow evolution, signs and symptoms such as occlusion alterations and tooth loosening may arise over time. Severe complications implying pathological bone fractures, an extensive development in the posterior area of the maxilla and mandible, could eventually influence breathing, and in rare cases, facial paralysis and malignant transformation could occur [11,12]. Diagnosing fibrous dysplasia in clinical settings can be challenging, particularly when the lesion is located in the maxilla or mandible. In these areas, the radiological and histological features may differ from those seen elsewhere, suggesting a possible dermal origin [13]. Panoramic radiography is usually the first imaging step and often reveals a ground-glass-like appearance without clear borders. Early lesions tend to be radiolucent, but as they progress, they develop a mixed radiolucent–radiopaque pattern and may eventually become predominantly radiopaque. CT scans can show three main patterns: a ground-glass-like image in more than 56% of cases, a uniformly dense appearance in about 23%, and a cyst-like aspect in roughly 21% [14]. Any noticeable change in CT characteristics might indicate increased mitotic activity and a potential malignant transformation [15]. A biopsy remains essential for confirming the diagnosis and ruling out other conditions, although microscopic evaluation can be difficult because some features overlap with those of other bone pathologies [16].

The frequency of sarcomatous transformation in fibrous dysplasia is reported to be less than 1% of cases (monostotic/polyostotic) to 4% (McCune–Albright/Jaffe–Lichtenstein syndromes). The most common malignant transformation of fibrous dysplasia is osteosarcoma, followed by fibrosarcoma, chondrosarcoma, and malignant fibrous histiocytoma. A painless swelling is usually the initial symptom of maxilla-facial fibrous dysplasia, followed by pain and paresthesia, which could suggest a malignant transformation of this entity. The symptoms change from a painless expansion of the affected area into a painful one, followed by paresthesia as the mandibular tumor invades the mandibular canal and affects the inferior alveolar nerve. The majority of the cases presented in the scientific literature have, as an etiologic factor responsible for the malignant transformation, the radiation treatment used for the initial treatment of fibrous dysplasia [17,18]. Our report shows an exceptionally rare example of spontaneous malignant transformation of FD into secondary chondrosarcoma in a non-irradiated patient. The other etiological factors in case of malignant degeneration are still unclear.

Chondrosarcoma is a malignant tumor with a cartilaginous component that rarely affects the mandible; most of the reports discuss a higher incidence in the maxilla [19]. Nevertheless, the present case involves a malignant transformation of fibrous dysplasia. The tendency of malignant transformation in patients without previous irradiation history is rarely reported, and generally, the prognosis of these cases is poor [20]. Lower survival rates were reported in patients who developed sarcomas from fibrous dysplasia compared to those diagnosed with primary sarcomas. In these cases, an accurate diagnosis and complete surgical resection would be the appropriate treatment option [21].

Taking into consideration the malignant transformation of fibrous dysplasia without previous radiation treatment and outlining the rare characteristics of this reported case and the fact that, in the scientific medical literature, only one other case of monostotic fibrous dysplasia localized in the mandible that underwent a malignant transformation to chondrosarcoma is described, the aim of the present case report is to share our experience in this rare case related to the management of chondro-sarcomatous transformation of fibrous dysplasia after it was surgically removed twice. By presenting the data related to the clinical aspects and the surgical management, it is important to assess the long-term outcomes of the treatment in case of malignant transformation of fibrous dysplasia.

This case report has been prepared in accordance with the CARE guidelines for case reports (https://www.care-statement.org/).

## 2. Case Report

### 2.1. Examination

**Figure 1:**
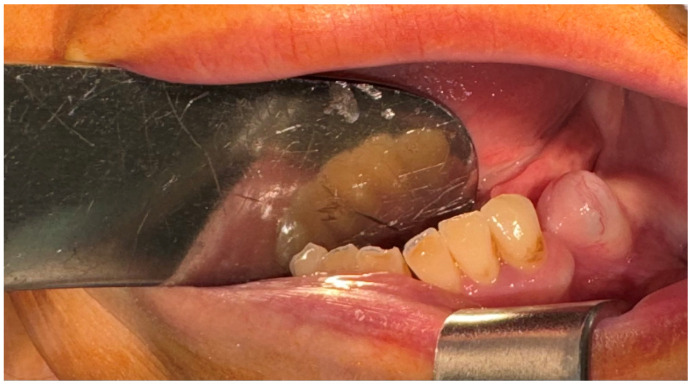
The initial aspect of the gingival tumor.

A 57-year-old female patient was referred by the dentist to the Oral and Maxillofacial Surgery Department of Emergency Hospital Timisoara following the identification of a gingival tumor in the third quadrant, being localized in the area of the second inferior premolar and first molar (Figure 1).

The patient noticed a gingival mass localized on the left side of the mandible, which was painless with an evolution of approximately 6 months, and presented for a consultation with her dentist, being afterwards referred to the maxillofacial surgery department. During the surgical consultation, the intraoral examination revealed a raised, painless, and tender-at-palpation tumor localized on the inferior mandibular left alveolar crest without extending into the vestibule or lingual region which had a normal color and appearance similar to that of the surrounding gingival mucosa.

**Figure 2:**
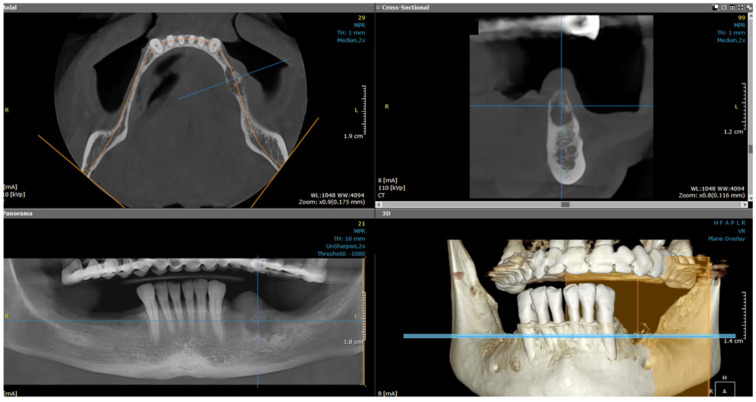
The CBCT aspect of the tumor: a 4 cm osteolytic area in the left mandibular body, distal from tooth 3.3. The osteolytic lesion appeared as radiolucent with coexisting radiopaque areas.

After the clinical intraoral examination, a mandible Cone Beam CT (CBCT) was performed (Figure 2). The outcome of this radiologic examination revealed a 4 cm osteolytic area in the left mandibular body, distal from tooth 3.3. The osteolytic lesion appeared as radiolucent with coexisting radiopaque areas. Nevertheless, no other subsequent similar radiopaque changes were identified in the surrounding tissue.

### 2.2. First Surgical Intervention

**Figure 3:**
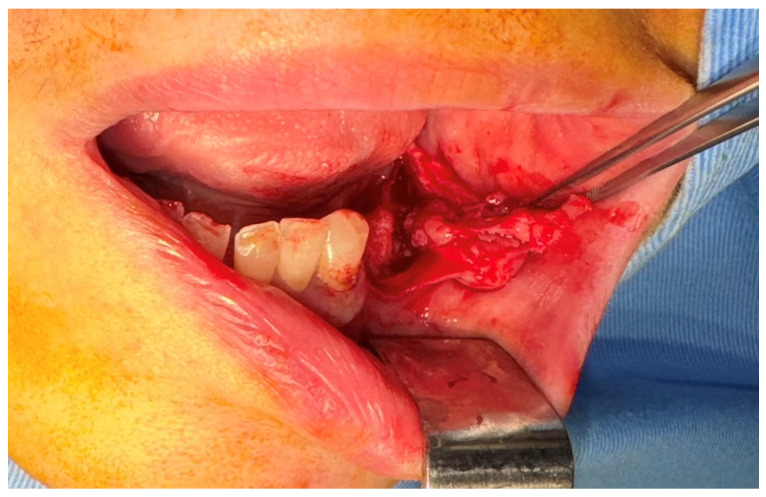
The aspect of the tumor before surgical removal.

**Figure 4:**
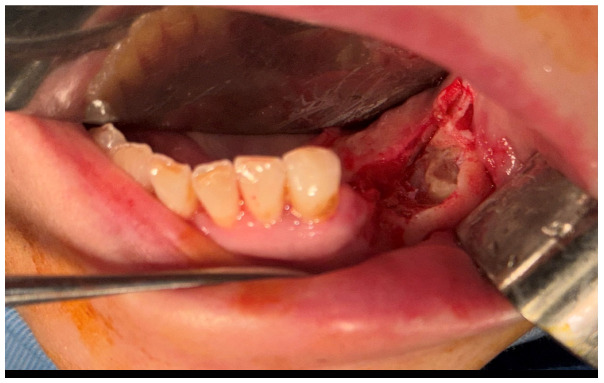
The aspect of the modified mandibular bone under the gingival tumor.

**Figure 5:**
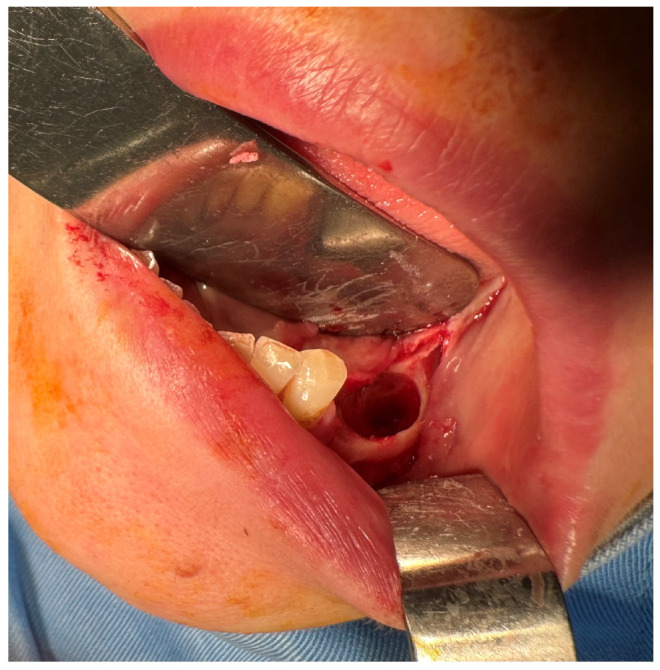
The mandibular bone aspect after surgery.

**Figure 6:**
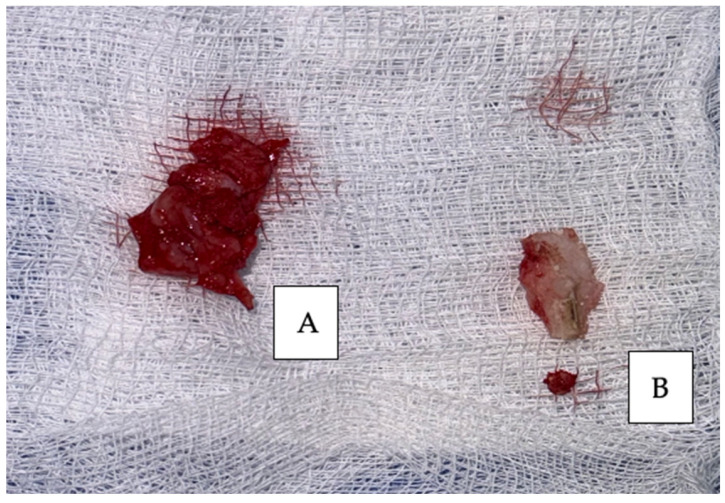
Surgical specimens removed: (**A**) gingival tumor and (**B**) modified mandibular bone.

After the clinical and paraclinical examination, the patient was placed under general anesthesia (intravenous type) performed with local anesthesia (troncular peripheric anesthesia of inferior alveolar nerve and lingual nerve) and local anesthesia infiltration for the buccal nerve. A modified horizontal incision was performed on the alveolar crest distal from the lower left canine to the retromolar triangle. This incision circumscribed the gingival tumor with 5 mm safety margins. Additionally, 2 vertical oblique incisions were made, medial to the canine and in the retromolar triangle area. A muco-periostal flap was raised and the gingival tumor was surgically removed (Figure 3). The modified mandibular bone aspect was exposed and with the piezotome under local irrigation, the pathologic bone was surgically removed (Figure 4, Figure 5 and Figure 6). The modified tissues that were removed were sent for histopathologic analysis.

**Figure 7:**
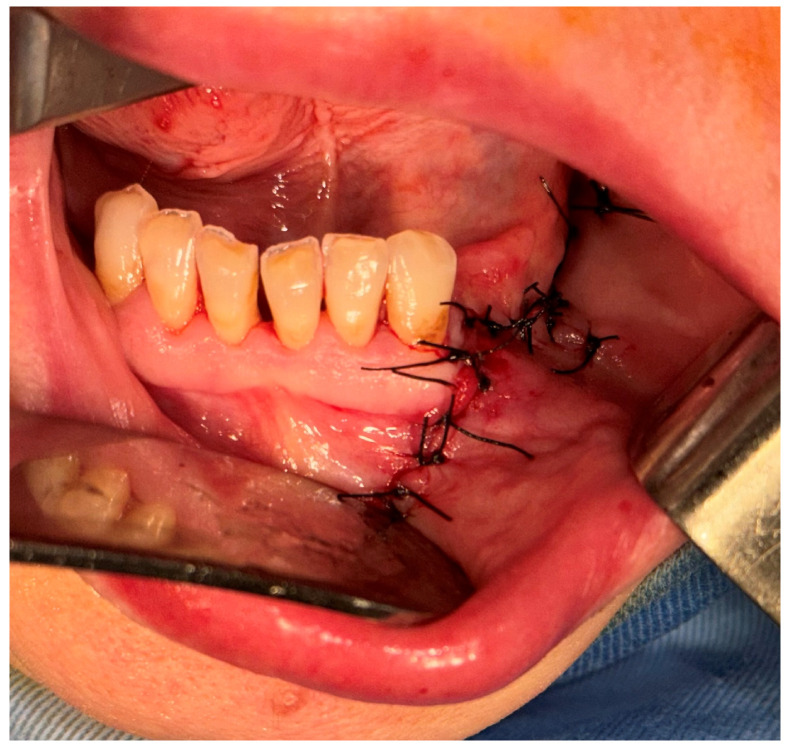
Sutures of the muco-periostal flap.

Interrupted suture of the muco-periostal flap was performed (Figure 7).

### 2.3. First Histopathologic Examination

**Figure 8:**
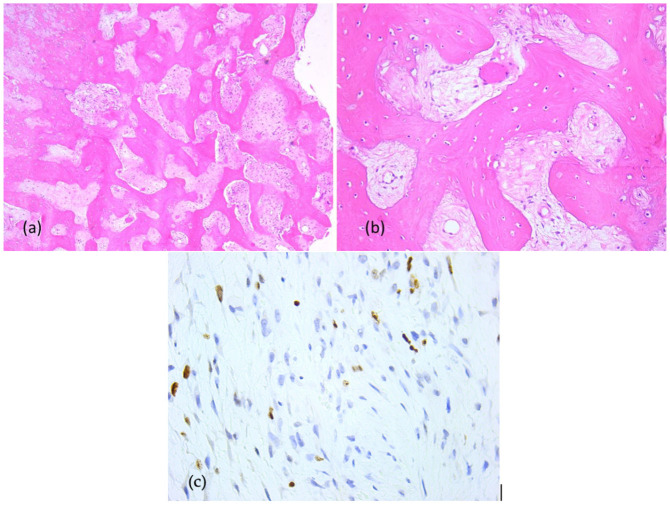
Fibrous dysplasia (first presentation), microscopic aspects in hematoxylin-eosin staining: Branching and anastomosing irregular trabeculae of woven bone with no conspicuous osteoblastic rimming and fibrous stroma with bland spindle cells, without cytologic atypia (**a**) 5× objective; (**b**) 20× objective; (**c**) Ki67 index: 35%.

Primary probe processing was performed on the paraffin-embedded tissues specimens processed according to a standard histology technique. The probes were cleaned, dried, clarified, and imbedded in paraffin. The Thermo Shandon standardized inclusion automat (Thermo Fisher Scientific Inc., Gothenburg, Sweden) was utilized for the inclusion stage. The Shandom ME microtome was used to perform sectioning. Two slices, each measuring around 3 to 5 μm in thickness, were cut from each paraffin block. Hematoxylin–eosin staining of one set of sections was performed using the Leica automatic system on a regular basis to verify the clinical diagnosis. Sections stained morphologically with hematoxylin–eosin were analyzed using the Zeiss Axiocam 506 (Zeiss, Jena, Germany) and Nikon AY260 microscopes (Nikon Europe B.V., Amstelveen, The Netherlands). Both microscopes are equipped with a real-time imaging system and software for the digital analysis of microscopic images. The Ki67 score was 35%. The initial histopathologic diagnostic was fibrous dysplasia (Figure 8).

The sutures were removed after 10 days, and the patient was scheduled for regular check-ups.

### 2.4. Second Surgical Intervention

**Figure 9:**
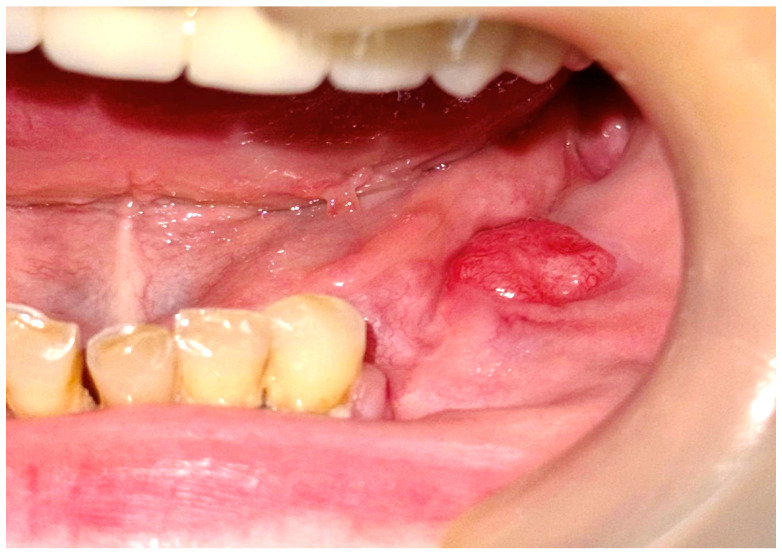
The clinical aspect of the gingival tumor.

**Figure 10:**
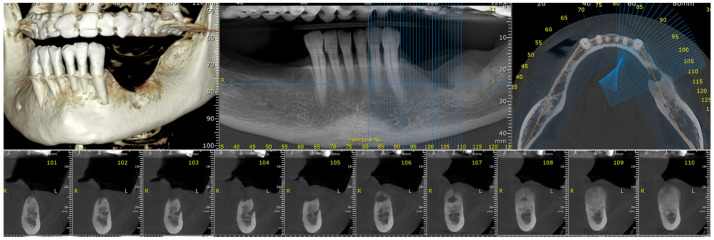
The radiologic aspect of the gingival tumor on CBCT: a 5 cm osteolytic area in the left mandibular body, distal from tooth 3.3. The osteolytic lesion appeared as radiolucent with coexisting radiopaque areas.

After 3 months, the patient presented in the clinic with hypoestesia of the inferior left lip and mentonier area. At the clinical intraoral examination, in the left mandibular area where the first surgical intervention was performed, a new gingivo-alveolar tumor was diagnosed (Figure 9 and Figure 10).

**Figure 11:**
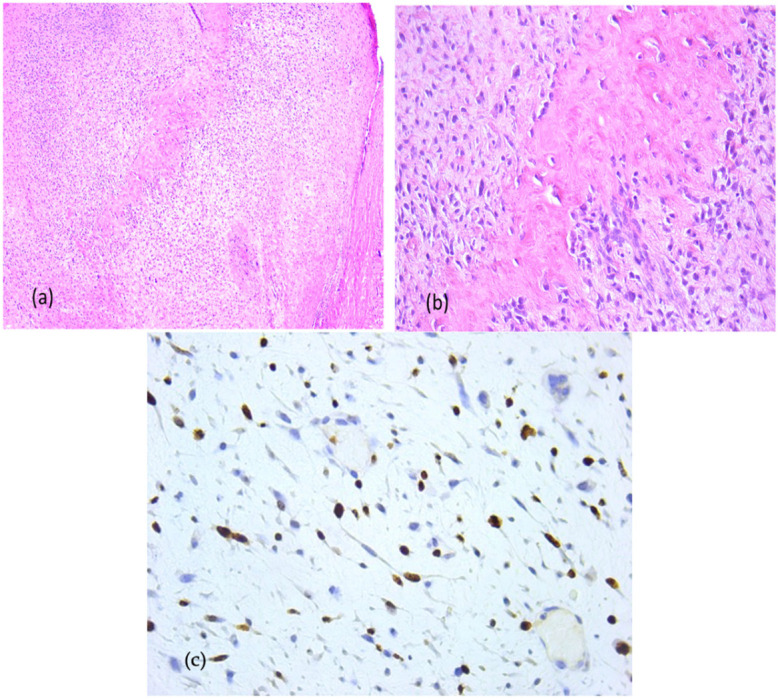
Fibrous dysplasia with cytological atypia, microscopic aspects in hematoxylin-eosin staining: Anastomosing irregular trabeculae of woven bone with no conspicuous osteoblastic rimming and dense fibrous stroma with irregular spindle cells, focal with minimally cytologic atypia and mitoses, (**a**) 5× objective; (**b**) 20× objective; (**c**) Ki67 index: 60%.

Due to the osseous extension, a second surgical intervention similar to the first one was performed in order to remove the gingival tumor and incisional biopsy of the mandibular modified bone. The second histopathologic diagnosis was fibrous dysplasia with myxoid component. The Ki67 score was 60% (Figure 11).

**Figure 12:**
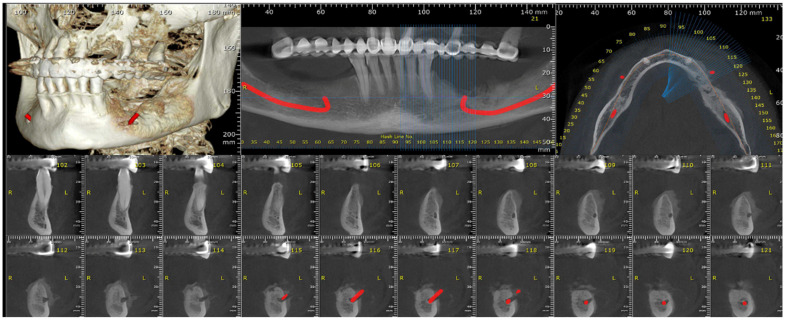
The radiologic aspect of the mandibular bone on CBCT.

After the second surgical intervention, the case had a fulminant evolution characterized by a rapid growth in the size of the tumor and exacerbated pain, especially during night, and was non-responsive to AINS. A CBCT and head computer tomography (CT) were also performed (Figure 12).

**Figure 13:**
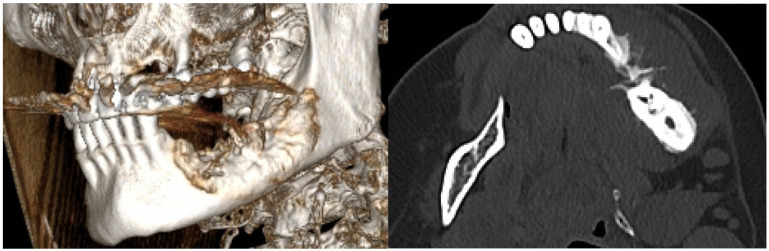
The radiologic aspect of the mandibular bone on CT: a lytic-sclerotic lesion affecting the left mandibular body with an extension of 38 mm, exhibiting a spiculiform periosteal reaction and extension into the periosteum.

The head CT revealed a lytic-sclerotic lesion affecting the left mandibular body with an extension of 38 mm, exhibiting a spiculiform periosteal reaction and extension into the periosteum (Figure 13).

### 2.5. Third Surgical Intervention

**Figure 14:**
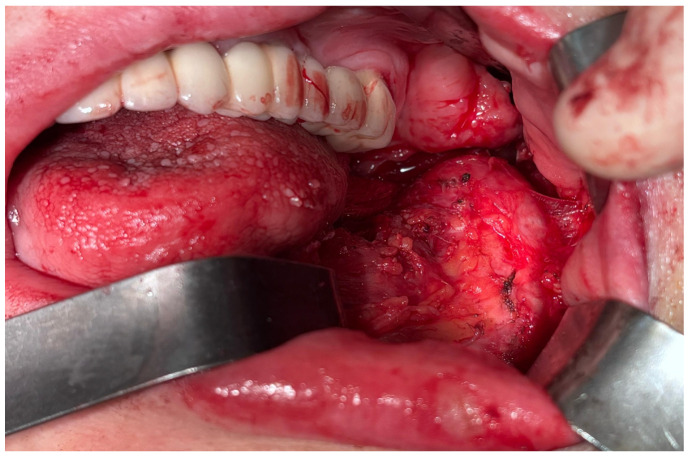
Aspect during the surgery.

**Figure 15:**
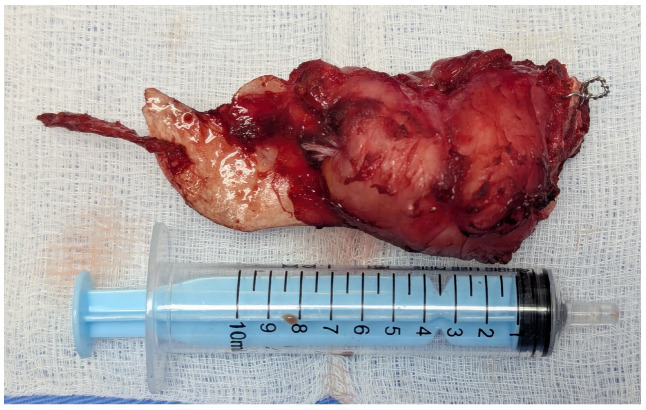
The resected tumor.

Taking into consideration all the clinical and radiological aspects of the mandibular bone, a malignant transformation was taken into consideration. Under general anesthesia, delivered intravenously and completed with local anesthesia—troncular peripheric anesthesia of the inferior alveolar nerve and lingual nerve and local anesthesia for the buccal nerve—a partial resection of the mandibular bone with surrounding soft tissues was performed from the inferior left canine up to the gonion (Figure 14 and Figure 15).

**Figure 16:**
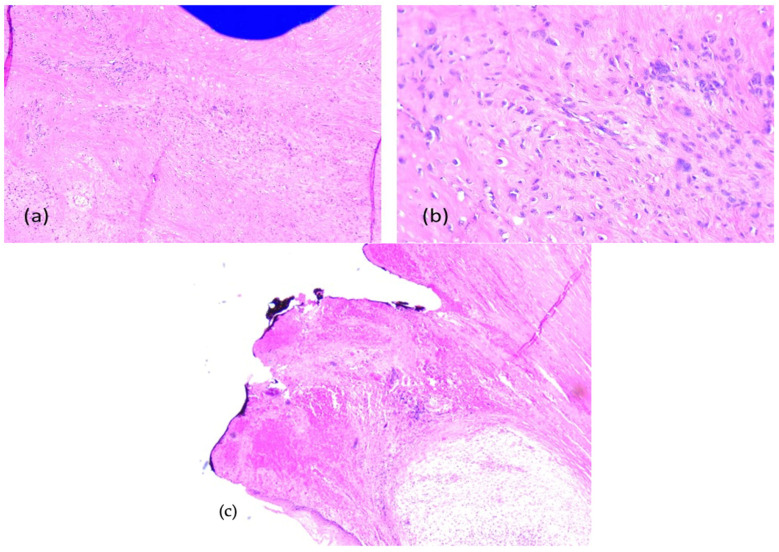
Fibrous dysplasia with malignant transformation to chondrosarcoma, microscopic aspects in hematoxylin-eosin staining: chondroid and spindle cells, uni-/bi- or multinucleated, with high pleomorphism and atypical mitoses, (**a**) 5× objective; (**b**) 20× objective; (**c**) tumor-free margins.

**Figure 17:**
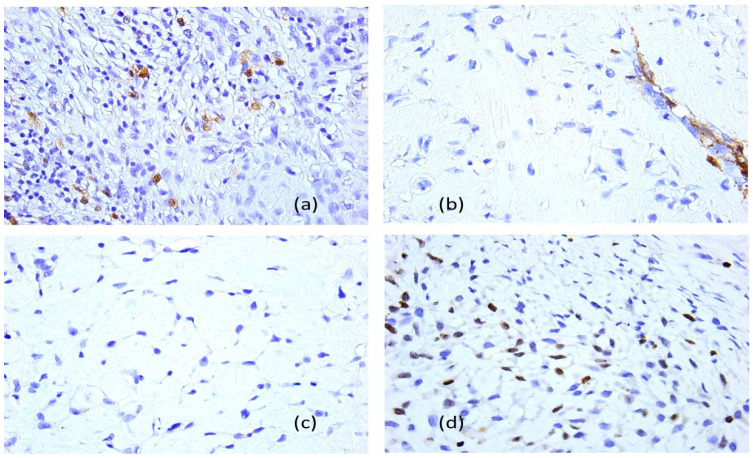
Fibrous dysplasia with malignant transformation in chondrosarcoma (the third presentation), immunohistochemical profile, 40× objective: (**a**) anti-S100 protein antibody, positive reaction; (**b**) anti-smooth muscle actin antibody, negative reaction, with positive internal control on blood vessels; (**c**) anti-epithelial membrane antigen–antibody, negative reaction; (**d**) anti-Ki 67 antibody, Ki67 cell proliferation index of 70%.

The immunohistochemistry was performed through a fully automated and standardized procedure using a Leica Bond-Max auto-stainer (Leica Biosystems, Newcastle upon Tyne, UK): CD34-negative reaction, vimentin-positive reaction, S100 protein-negative reaction, and desmin-negative reaction. Sections stained morphologically with hematoxylin–eosin and immunohistochemically were analyzed using the Zeiss Axiocam 506 (Jena, Germany) and Nikon AY260 microscopes (Nikon Europe B.V., Amstelveen, The Netherlands). Both microscopes are equipped with a real-time imaging system and software for the digital analysis of microscopic images. The third histopathologic result revealed the diagnosis of a fibrous dysplasia with malignant transformation into chondrosarcoma of high grade III. The tumor extended to the gingival mucosa, and it was removed completely, with no tumoral invasion in the resection margins (Figure 16 and Figure 17).

Three weeks after surgery, during the interdisciplinary approach to the case, the oncologist decided to start adjuvant radiotherapy, and the dosage prescriptions that were used were 66 Gy/33fx.

For 1.5 years, the patient came for regular follow-ups in which clinical and radiologic assessments were performed, and, until the present, there have been no signs of local tumor recurrence so far.

## 3. Discussion

The diagnosis of fibrous dysplasia is difficult because there are no distinguishing signs, and the majority of patients complain of several non-specific symptoms such as pain, swelling, or facial distortion. It is known that this condition occurs more commonly in the maxilla than in the mandible and that females are more often affected [22,23].

Health care professionals like dentists and oral and maxillo-facial surgeons should be aware of this pathology in order to avoid misdiagnosis; comprehensive clinical, radiographic, and histopathologic examinations should be performed to identify whether fibrous dysplasia or its potential malignant transformation exists. Malignant transformation is an uncommon evolution. The probability of malignant transformation is anticipated to be between 1% and 5.7% of cases for the monostotic type, and approximately 4% for the polyostotic form [24,25]. Clinicians should take into consideration this risk because it will influence their surgical approach and further follow-up plans. The exact malignant transformation of craniofacial fibrous dysplasia is difficult to estimate; until now, few reports have been mentioned in the literature [26].

The most prevalent histologic type of fibrous dysplasia malignant transformation is osteosarcoma, followed by fibrosarcoma, chondrosarcoma, and malignant fibrous histiocytoma. Our case presents a rare evolution of fibrous dysplasia towards malignancy into one of the rarest types, chondrosarcoma. Only a few cases were reported in the literature of malignant transformation of fibrous dysplasia and the development of chondrosarcoma with localization at the mandible. Sun et al. [23] revealed a predominance of the malignant transformation of FD in the case of females. However, this type of transformation can occur in monostotic and polyostotic cases as well, with a slightly more increased incidence in polyostotic FD, specifically in patients diagnosed with Mazabraud syndrome and MAS [27]. The incidence of chondrosarcoma in the context of malignant transformation of FD affecting the mandible in the craniofacial region has been reported in only a few cases. Only one identified case of malignant transformation of a monostotic form of FD in the mandible, also a female, was reported in the literature [28]. In the other three cases of chondrosarcoma development in the mandible, the described form of FD was the polyostotic one in the context of patients diagnosed with MAS [29,30,31].

It is well known that radiotherapy should not be the first elected treatment for fibrous dysplasia since it can increase the chance of developing sarcoma [32]. This was the reason that led us to adopt a strategy of watchful waiting the case after the first surgical intervention. For malignant transformation, the average time is 11.3 years and the mean age is 36.4 years [26]. In our case, the period from the second surgical intervention until the malignant transformation was approximately 1 year.

Malignant transformation of fibrous dysplasia appears to arise from a wide range of genetic disturbances rather than a single defining alteration, involving losses of important tumor-suppressor regions, abnormal activation of growth-related genes, and various chromosomal imbalances that recur across many cases. Large analyses of abnormal karyotypes have shown that several chromosomal regions are repeatedly disrupted, and that gains or losses of specific chromosomes—such as extra copies of chromosome 1 or deletions affecting chromosomes 9, 10, 13, and 17—occur with notable frequency. Among these genetic changes, alterations affecting the RB1 and TP53 genes stand out as particularly significant: damage to TP53 interferes with normal DNA repair and undermines cellular safeguards, while loss of RB1 disrupts the cell-cycle controls that normally keep cell division in check. Together, defects in these two critical pathways are thought to play a central role in driving the development and progression of chondrosarcoma [33].

The prognosis of the malignant transformed fibrous dysplasia is poor. The recurrences after the surgery or oncologic treatment were noted within 5 years. The 5-year survival rate for head and neck primary osteosarcoma was around 60%, but in cases of fibrous dysplasia with malignant transformation, the overall survival rate is around 30% after 3 years and 20% at 5 years [34]. This discrepancy points out that malignant transformation in the case of fibrous dysplasia has a more aggressive evolution.

The studies show that patients with mandibular localization of a fibrous dysplasia with a malignant transformation had a better prognosis compared to other locations due to better surgical access in the mandibular area, facilitating a complete removal with tumor free margins [25]. On the other side, the extension of the surgical resection is limited by the unclear limits of the malignant fibrous dysplasia and the vicinity of the vital neurovascular surrounding structures.

The effects of adjuvant oncologic treatment such as radiotherapy and chemotherapy for malignant transformed fibrous dysplasia are still unclear. It can be utilized to increase progression-free overall survival and improve local control of these resected malignancies. In the scientific literature, the ionizing radiation doses ranged from 4.6 to 70.4 Gy, with the majority of patients receiving a reported ionizing radiation dose between 40 and 50 Gy [35]. In combination with the rarity of malignant transformation in the case of fibrous dysplasia, the treatment efficacy and management of the case are important steps to improve the overall survival rate. Nevertheless, more cases need to be analyzed in order to estimate the impact on these malignancies in the head and neck area.

Regarding the imagistic evaluation, MRI was not performed, which we recognize as a limitation. The decision reflected the patient’s clinical presentation and the fact that CT already provided sufficient detail to characterize the lesion and guide management. There were no clinical or radiologic features at the time suggesting soft-tissue extension or other findings that would have prompted further imaging. Even so, MRI can offer complementary information, particularly when there is concern of more aggressive behavior or possible malignant transformation, as it allows for better assessment of marrow involvement and adjacent soft tissues. Future evaluations would benefit from incorporating MRI when there is any uncertainty regarding lesion activity or progression.

## 4. Conclusions

Non-radiation-induced malignant transformation of mandibular fibrous dysplasia is a very rare and challenging complication, as our case report shows. Its early recognition by clinicians and proper surgical treatment are the key factors to prevent further evolution in the case. Even though it is characterized by a bad prognosis, early surgical resection within free tumor margins is strongly advised. The risk of chondrosarcomatous transformation must be considered for all age and gender groups of patients diagnosed with fibrous dysplasia.

## Data Availability

The original contributions presented in the study are included in the article. Further inquiries can be directed to the corresponding authors.
